# Design of target-variable spraying system based on FAVD of fruit tree canopy

**DOI:** 10.3389/fpls.2025.1582664

**Published:** 2025-05-23

**Authors:** Shijie Jiang, Wenwei Li, Hao Ma, Kexin Wang, Zhe Du, Yongjun Zheng

**Affiliations:** ^1^ College of Agricultural Equipment Engineering, Henan University of Science and Technology, Luoyang, China; ^2^ College of Engineering, China Agricultural University, Beijing, China

**Keywords:** orchard, foliage area volume density (FAVD), uniform spraying, target-variable, air-assisted sprayer

## Abstract

**Introduction:**

Using air-assisted sprayer for chemical pesticide application is the main method for controlling pests and diseases in orchards. Target-variable spray based on canopy characteristics is an effective means to solve the problems of over-spraying, excessive residues, and environmental pollution.

**Methods:**

Foliage area volume density (FAVD), as it represents the number of pesticide targets in the canopy, can be used as a decision condition for variable spraying. Based on the previous FAVD detection method, this study developed a small target-variable sprayer based on FAVD, constructed a FAVD-spray rate control model, and conducted orchard experiments.

**Results:**

The experiment results showed that the targeted variable spray (TV) mode significantly improved deposition uniformity, and reduced ground loss and water consumption. For the TV model, the longitudinal variation coefficient was 11.42%, and the lateral variation coefficients were 55.27% (top layer), 58.80% (middle layer), and 43.15% (bottom layer), respectively. For the NTIV model, the longitudinal variation coefficient is 32.15%, and the lateral variation coefficients were 96.19% (top layer), 62.69% (middle layer) and 57.19% (bottom layer) respectively. In terms of ground and behind-canopy losses, the TV model reduced 79.78% and 73.54%, respectively, and saved 64.50% of water consumption.

**Discussion:**

Compared with the NTIV model, the TV model has small longitudinal and lateral coefficients of variation, the loss of droplets on the ground and behind-canopy is greatly improved, and it can significantly reduce the amount of water consumption. Target variable spraying based on FAVD can significantly improve the uniformity of droplet distribution in the canopy, reduce ground loss and environmental pollution, and provide a reference for the development of precision spraying technology in orchards.

## 1 Introduction

The primary method for controlling diseases and pests in orchards is spraying chemical pesticides. However, continuous, excessive, and rough spray has caused serious waste of pesticides and environmental pollution, and the effective utilization rate of pesticides is only 40% (Meshram et al., 2022). Target-variable spray based on fruit tree canopy characteristics is an effective means to solve pesticide waste and environmental pollution (Salcedo et al., 2020; Manandhar et al., 2020; Cai et al., 2019).

In the current study, canopy profile (Gu et al., 2020; Wei et al., 2023), canopy volume (Liu et al., 2024, Liu et al., 2022) and leaf wall area (LWA) (Chen et al., 2024; Sun et al., 2022) were mainly used as decision conditions for variable spray. The canopy profile refers to the projection of the external shape of the fruit tree canopy, which reflects the size and shape of the canopy. Jiang et al. (2016) developed an air-assisted target spra\yer based on ultrasonic sensors, which controls the number of nozzles activated according to the canopy profile, saving 30% of the pesticide solution. Using the canopy profile as a condition for targeted variable spray can reduce pesticide waste, but the effect of spraying for fruit trees with sparse canopies was not significantly improved (Li et al., 2023). Compared to the canopy profile, the canopy volume not only reflects the shape and size of the canopy in a two-dimensional plane but also represents its three-dimensional structure (Gu et al., 2021). Dou et al. (2022) developed a spraying control method based on canopy volume. Cai et al. (2019) used LiDAR point cloud data to represent the canopy grid volume, and designed a spray flow control model using a pulse-width modulation (PWM) duty cycle. The LWA can reflect the exposed leaf area in the actual pesticide spray zone (Liao et al., 2020), particularly in densely planted orchards, where it can effectively improve spray coverage and pesticide utilization efficiency. Xiao et al. developed a variable spray control model based on LWA for grape growth, significantly reducing pesticide use while ensuring uniform distribution of the pesticide solution on the canopy surface (Xiao et al., 2017; Gao et al., 2018). Hoevar et al. (2010) used color images captured by RGB cameras to extract the LWA region as a variable decision condition, which saved 23% of pesticides. Xue et al. (2020) used LiDAR to obtain LWA and built a fruit tree pesticide spray model based on LWA, conducted spraying experiments. However, while the canopy profile, canopy volume, and LWA can, to some extent, characterize the growth features of the canopy and reduce pesticide application when used as decision variables, these parameters either overlook or simplify the irregular, asymmetric, and porous structure of the canopy (Zeng et al., 2020; Narváez et al., 2016), leading to significant discrepancies between the calculated results and actual canopy parameters.

In study of canopy profile, canopy volume, or LWA, the typical method involves “sensor scanning/data collection—solving geometric equations—fitting canopy characteristics.” Although this method achieves variable spraying to some extent, the primary targets for the spray—fruit tree leaves—are mostly concentrated at the outer ends of branches, while the leaves inside the canopy are relatively sparse. Additionally, the density of leaves varies across different parts of the canopy. Using canopy volume and profile as decision-making criteria essentially overlooks the leaves as the main targets for spraying, thus failing to achieve true precision spraying.

Foliage area volume density (FAVD) refers to the total leaf area within a unit volume of the canopy (m²/m³). Compared to canopy profile and canopy volume, FAVD better represents the canopy’s growth characteristics and quantifies the tree crown’s density or sparsity (Mahmud et al., 2021; Sun and Liu, 2019). Previous studies have demonstrated that FAVD has a significant impact on droplet deposition within the fruit tree canopy and droplet drift behind the canopy (Jiang et al., 2022, Jiang et al., 2023). Using FAVD as a decision-making criterion for target-variable spraying not only ensures effective spraying of the canopy but also significantly reduces water usage and minimizes ground loss. Therefore, researching FAVD detection methods and constructing a spraying decision model based on FAVD holds practical significance.

In summary, based on previous methods of FAVD detection, this study develops small target-variable sprayer based on FAVD, design spraying control method based on FAVD, and establish FAVD-spray rate control model, orchard experiments are conducted. This study provides available methods to improve the utilization of pesticides, reduce droplet loss and environmental pollution, and references for the development of precision spray technology in orchards.

## 2 Materials and methods

### 2.1 Design of orchard target-variable sprayer

#### 2.1.1 Overall design scheme

**Figure 1:**
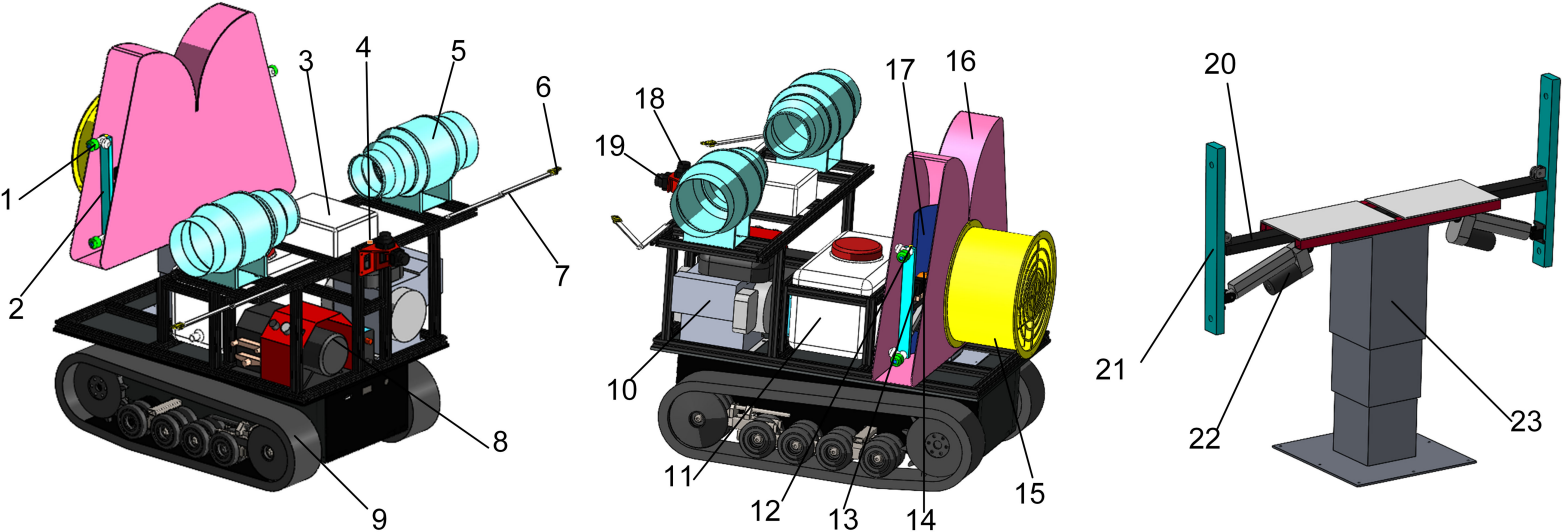
Orchard target-variable sprayer.

The overall scheme of the small target-variable sprayer is shown in **Figure 1**. It mainly includes the walking chassis, FAVD detection system, targeting system, automatic navigation system, air-assisted system, and spraying system. The chassis uses an electric crawler chassis powered by lead-acid batteries, along with a range extender to improve endurance. The FAVD detection system consists of excitation fan, folding arm support, and audio collection sensor. The targeting system includes the target actuator, target LiDAR, and pitch angle attitude sensor. The target actuator is designed symmetrically, consisting of lifting mechanism, support beam, swing arm mechanism, and linear actuator. The angle of the swing arm mechanism is adjusted by the linear actuator. Based on the results from the LiDAR and pitch angle attitude sensor, the position of the lifting and swing arm mechanisms is adjusted to achieve accurate targeting operations. The automatic navigation system includes navigation LiDAR and vehicle attitude sensor. The air-assisted system includes an axial flow fan, air duct, and deflector. The spraying system includes pump, tank, solenoid valve, proportional valve, nozzle body, and nozzle.

1. nozzle body and nozzle 2. target actuator 3. control cabinet 4. vehicle attitude sensor 5. excitation fan 6. audio collection sensor 7. folding arm support 8. pump 9. crawler chassis 10. range extender 11. tank 12. pitch angle attitude sensor 13. solenoid valve 14. proportional valve 15. axial flow fan 16. air duct 17. deflector 18. navigation LiDAR 19. target LiDAR 20. support beam 21. swing arm mechanism 22. linear actuator 23. lifting mechanism

#### 2.1.2 Control system

**Figure 2:**
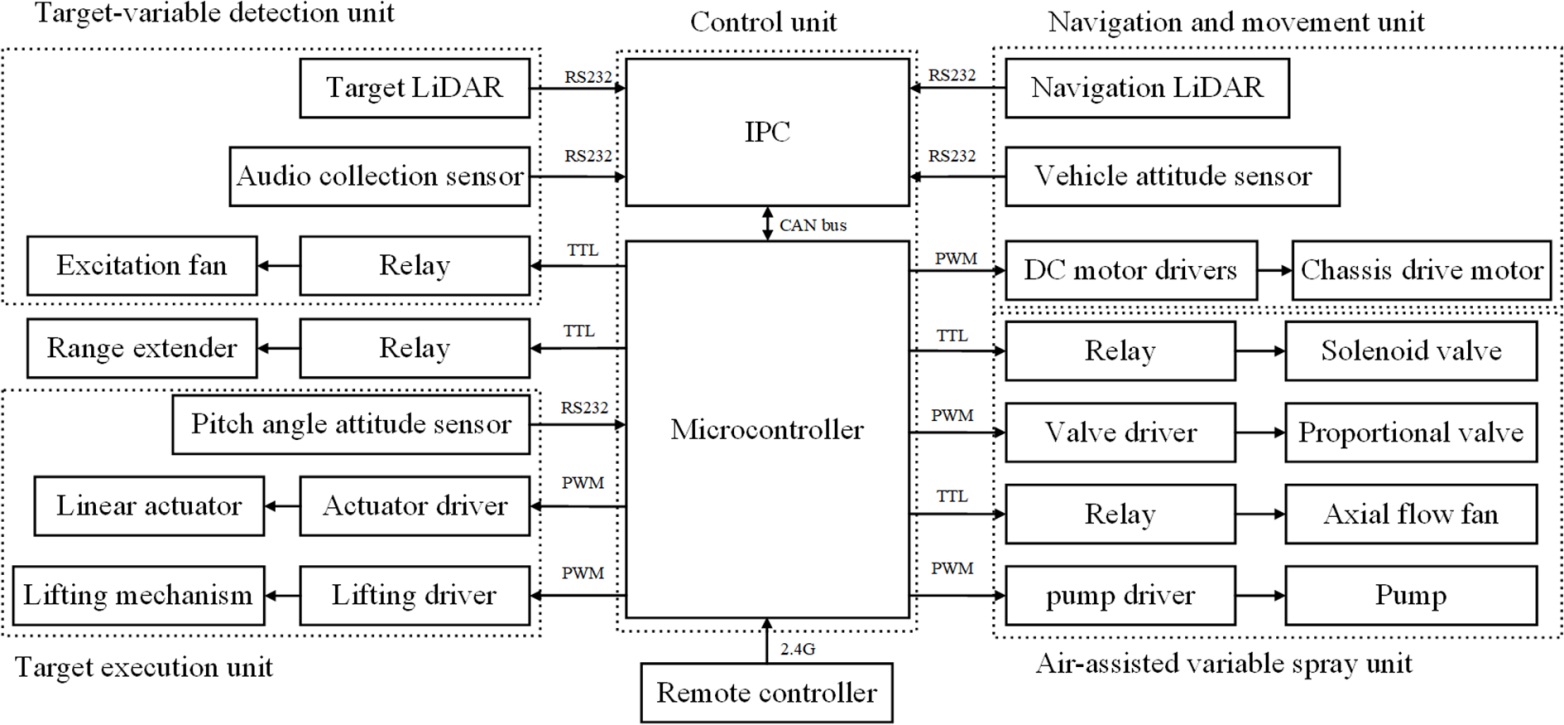
Overall scheme diagram of the control system.

The control system includes industrial control computer (IPC), microcontroller, remote control module, and drive module. **Figure 2** shows the overall scheme diagram of the control system, which is mainly divided into the control unit, target-variable detection unit, target execution unit, navigation and movement unit, and air-assisted variable spray unit.

The control unit includes a host computer and a slave computer. The host computer is an IPC (GK7000, Shenzhen Zhanmei Technology Co., Ltd.), which is responsible for processing signals from the navigation LiDAR, vehicle attitude sensor, target LiDAR and audio collection sensor, and sending the processed instructions to the slave computer. The IPC communicates with the sensors via an RS232 serial port and communicates with the slave computer via a CAN bus (Controller Area Network). The slave computer uses an STM32F103C8T6 microcontroller, which sends PWM signals (Pulse Width Modulation) to each driver to control the chassis motor, proportional valve, pump, linear actuator, and lifting mechanism. It sends TTL signals to relays to control solenoid valves, axial flow fans, range extender, and excitation fan. Additionally, the microcontroller communicates with the pitch angle attitude sensor via an RS232 serial port, obtaining real-time data from the sensor to achieve closed-loop control of the linear actuator.

#### 2.1.3 Working principle

**Figure 3:**
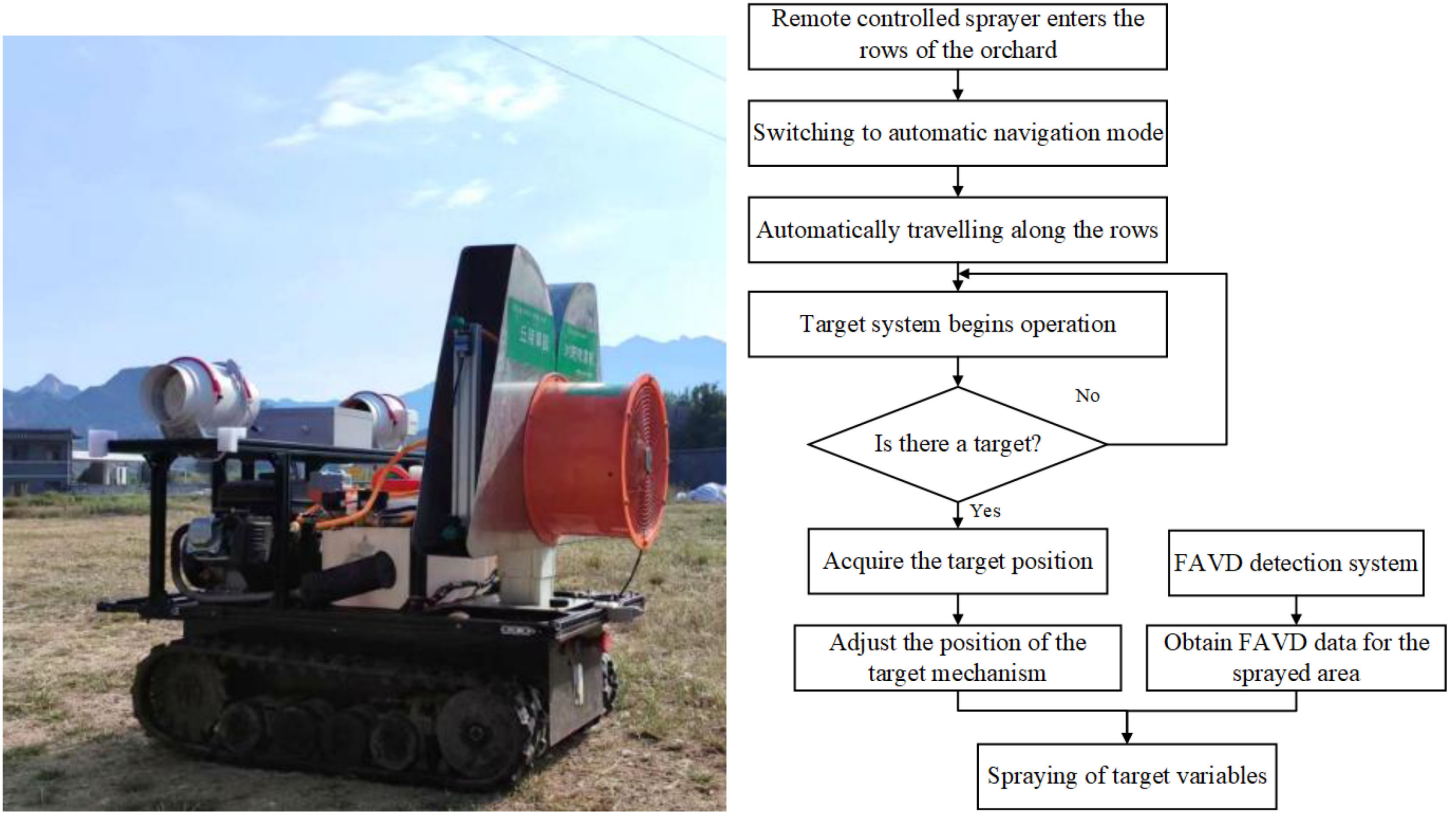
Working principle of the sprayer.

The sprayer nozzles are symmetrically arranged on both sides, allowing for easy switching between dual-side spray mode and single-side spray mode. The chassis incorporates inter-row auto-navigation system, which is operated by remote control for transferring, entering and exiting the garden, and turning in the ground, and can be switched to the auto-navigation mode with a single button during inter-row operation. The target system can adjust spraying based on the presence and location of target, achieving the function of “spray when there is a target, stop when there is none, and adjust based on target position.” The FAVD detection system collects the FAVD of the fruit tree canopy and uses it as a decision criterion to implement variable spraying, following the principle “more leaves, more spray; fewer leaves, less spray.” The basic workflow of the sprayer during fruit tree spraying operations is as follows (**Figure 3**):

- Remote controlled sprayer enters the rows of the orchard.
- Switch to automatic navigation mode with one button, and the sprayer automatically travels along the inter-row path.
- The target system begins operation by detecting the presence or absence of a target.
- When a target is detected, the system further acquires the target’s position, the target mechanism automatically adjusts its position, and the FAVD detection system begins to work, gathering the FAVD of the area to be sprayed.
- After adjusting the target mechanism position and obtaining FAVD data, and the target-variable spraying.

### 2.2 Estimation of FAVD in fruit tree canopy based on excitation audio

**Figure 4:**
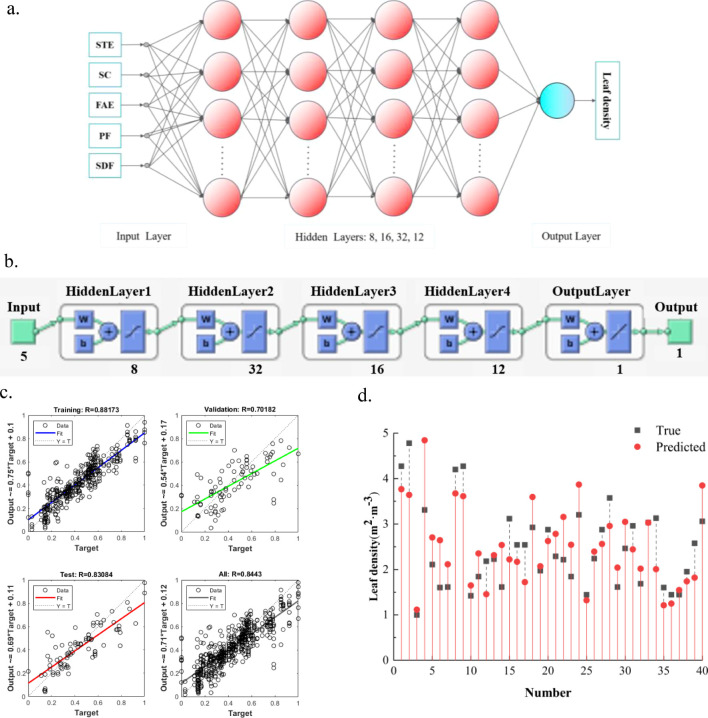
Construction of the FAVD estimation model based on the BP neural network. **(a)** Schematic diagram of the FAVD estimation model structure. **(b)** Model Structure. **(c)** Model Fitting Results. **(d)** Model Estimation Performance.

For the lack of automatic FAVD measurement methods, the high environmental requirements of conventional methods, and the large recognition error, the FAVD estimation method based on excitation audio was proposed by using the characteristics of the affected sound of fruit tree canopies (Li et al., 2024). Pearson correlation coefficients and variance analysis were used to determine the correlation between FAVD and excitation audio feature parameters. Five audio feature parameters were extracted for the construction of the FAVD estimation model, namely Short-time Energy (STE), Spectral Centroid (SC), Frequency Average Energy (FAE), Peak Frequency (PF) and Standard Deviation of Frequency (SDF). The FAVD estimation model was constructed based on a BP neural network (**Figure 4**). The overall correlation coefficient was 0.84, the root mean square error was 0.73, and the mean absolute error was 0.53. Both the model fitting and estimation performance met the required standards.

### 2.3 Construction of variable spraying control model based on FAVD

According to NYT 992-2006 (Agricultural Machinery of Standardization Administration of China, 2006), the standard for low-dose spraying in fruit trees is that the number of droplets per square centimeter on the leaves must be no less than 25, which corresponds to 2.5×10^5^ droplets per square meter. On this basis, this study designs a variable spraying control model based on FAVD.

The first step is the calculation of the effective spraying demand per unit volume of the fruit tree canopy. Assuming the FAVD is *ρ_favd_*, and the spraying is required on both the front and back of the leaves, the leaf area per unit volume is 2*ρ_favd_*. Therefore, the number of droplets required per unit volume can be calculated by Equations 1


(1) $$n=2\times 2.5\times 10^{5}\cdot \rho_{favd}=5\times 10^{5}\cdot \rho_{favd}$$

Where *n* is the number of droplets required per unit volume.

Simplifying the droplet to a spherical shape, the required amount of spray liquid per unit volume is shown in Equations 2:


(2) $$Q_{1}=5\times 10^{5}\cdot \rho_{favd}\cdot \frac{1}{6}\pi D_{v}^{3}$$

Where *Q*_1_ is the amount of spray liquid required per unit volume, and *D_v_* is the droplet diameter.

According to the literature (Wang, 2018), the relationship between the droplet diameter *D_v_* and the spray pressure satisfies Equations 3:


(3) $$D_{v}=C_{v}P^{-\frac{1}{3}}$$

Where *C_v_* is a constant related to the nozzle, which depends on factors such as the surface tension coefficient of the liquid and air density, in this study, the nozzle selected was Teejet TP6503, and the value of *C_v_* was calculated based on the *C_v_* measurement method provided in the literature (Wang, 2018), combined with the particle size spectra of the TP6503 nozzle. *C_v_* is taken as 3.0×10^-4^, and *P* is the spray pressure.

During the process of droplets traveling from the nozzle to the canopy, some droplets may evaporate or be lost. Therefore, the amount of spray liquid expelled by the nozzle satisfies Equations (4):


(4) $$Q=k\cdot Q_{1}+Q_{2}$$

Where *Q* is the amount of spray liquid expelled by the nozzle, *Q*_2_ is the liquid loss, and *k* is the correction factor, which is taken as 2 in this study.

*Q*_2_ is related to the distance *L_d_* between the nozzle and the canopy as shown in Equations (5):


(5) $$Q_{2}=\delta L_{d}\cdot Q$$

Where *δ* is a constant greater than 0, and in this study, *δ* is taken as 0.1.

The relationship between the amount of spray liquid *Q* expelled by the nozzle and the nozzle flow rate *q* is given by Equations (6):


(6) $$\begin{array}{l} Q=q\cdot t\\ t=\frac{L_{v}}{v} \end{array}$$

Where *t* is the time required for the nozzle to expel the spray liquid *Q*, which is related to the speed *v* of the sprayer, and *Lv* is the distance traveled by the sprayer during time *t*.

Therefore, the spray flow rate of the nozzle satisfies Equations(7):


(7) $$q=\frac{5\times 10^{5}\cdot kv\;\rho_{\;favd}\pi C_{v}^{3}}{6\left(1-\delta L_{d}\right)PL_{v}}$$

Under a certain pressure and frequency range, the spray flow rate *q* of the nozzle is linearly related to the PWM duty cycle *x*, as follows Equations (8):


(8) q=ax+b

Combining Equations (7) and Equations (8), the relationship between x and FAVD meets Equations (9):


(9) $$x=\frac{5\times 10^{5}\cdot kv\;\rho_{\;favd}\pi C_{v}^{3}}{6a\left(1-\delta L_{d}\right)PL_{v}}-\frac{b}{a}$$

Note: The model is suitable for wind speed less than 3.5m/s, temperature 15-25°C, and relative humidity less than 40%.

### 2.4 Experimental scheme

#### 2.4.1 PWM-Based calibration of spray flow rate

The experiment was conducted on September 18, 2022, at the orchard base of Fuyu Investment Co., Ltd. in Fuping County, Baoding City, Hebei Province. The purpose of the experiment was to obtain the relationship between the proportional valve’s opening angle and the nozzle flow rate. The main experimental equipment used included an electronic scale (for weighing, measuring error ±0.1g), a smartphone (for timing, measuring error 0.01s), and a sprayer. The proportional valve’s opening angle was controlled by PWM duty cycle signal sent by the microcontroller. The frequency of the PWM signal is set to 20 Hz. A duty cycle of 0-100% corresponds to the opening angle of the proportional valve from 0-100%, where 0% indicates the valve is fully closed and 100% indicates it is fully open. The experimental method is as follows:

- Adjust the opening angle. For the first set of experiments, the opening angle was set to 5%, and the water flow from the left and right nozzles was collected separately using water buckets (the empty bucket was weighed before the experiment begins).
- Open the solenoid valve and the pump. The spray pressure was set to 0.5 MPa, and water was continuously collected for 60 seconds.
- Close the solenoid valve and the pump. Use the electronic scale to weigh the water bucket and calculate the net amount of water collected (the total weight of the bucket minus the weight of the empty bucket).
- Repeat the above process three times for each opening angle and take the average value.
- After completing one set of experiments, adjust the opening angle and repeat the process. The opening angle is first increased by 5%, and the overall pattern of spray flow rate was observed.
- Based on the overall variation pattern, select an appropriate range of opening angles and conduct supplementary experiments. The supplementary experiments increase by 2% per set for a total of 30 sets.

During the experiment, the opening angles of the proportional valves on both sides were controlled simultaneously, and the average water flow from both nozzles was taken as the spray flow rate corresponding to each duty cycle signal. Based on the above experimental results, a one-factor linear fitting method was used in this study to model the correspondence between duty cycle and spray flow rate of the nozzle.

#### 2.4.2 Orchard spraying effect verification

##### 2.4.2.1 Experiment location

**Figure 5:**
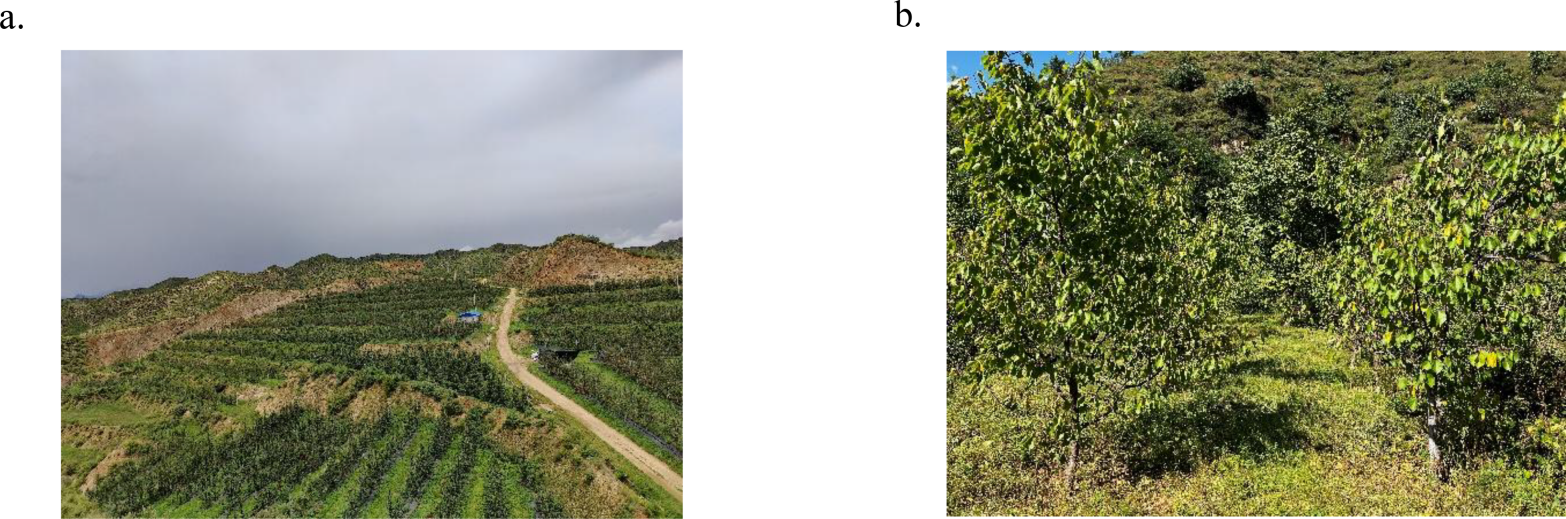
Orchard environment. **(a)** Topography of the experimental orchard. **(b)** Apricot orchard.

The experiment was conducted from September 20 to September 28, 2022, in the apricot orchard base of Fuyu Investment Co., Ltd. in Fuping County, Baoding City, Hebei Province, China. The experimental orchard is shown in **Figure 5**, and the planting parameters are listed in **Table 1**. Row spacing, plant spacing, plant height, and other parameters were randomly measured ten times in the orchard using a tape measure, and the average value was taken.

**Table 1:** Orchard planting parameters.

Category	Apricot orchard
Row spacing	3.9 m
Plant spacing	2.9 m
Plant height	2.8 m

##### 2.4.2.2 Experiment design

**Figure 6:**
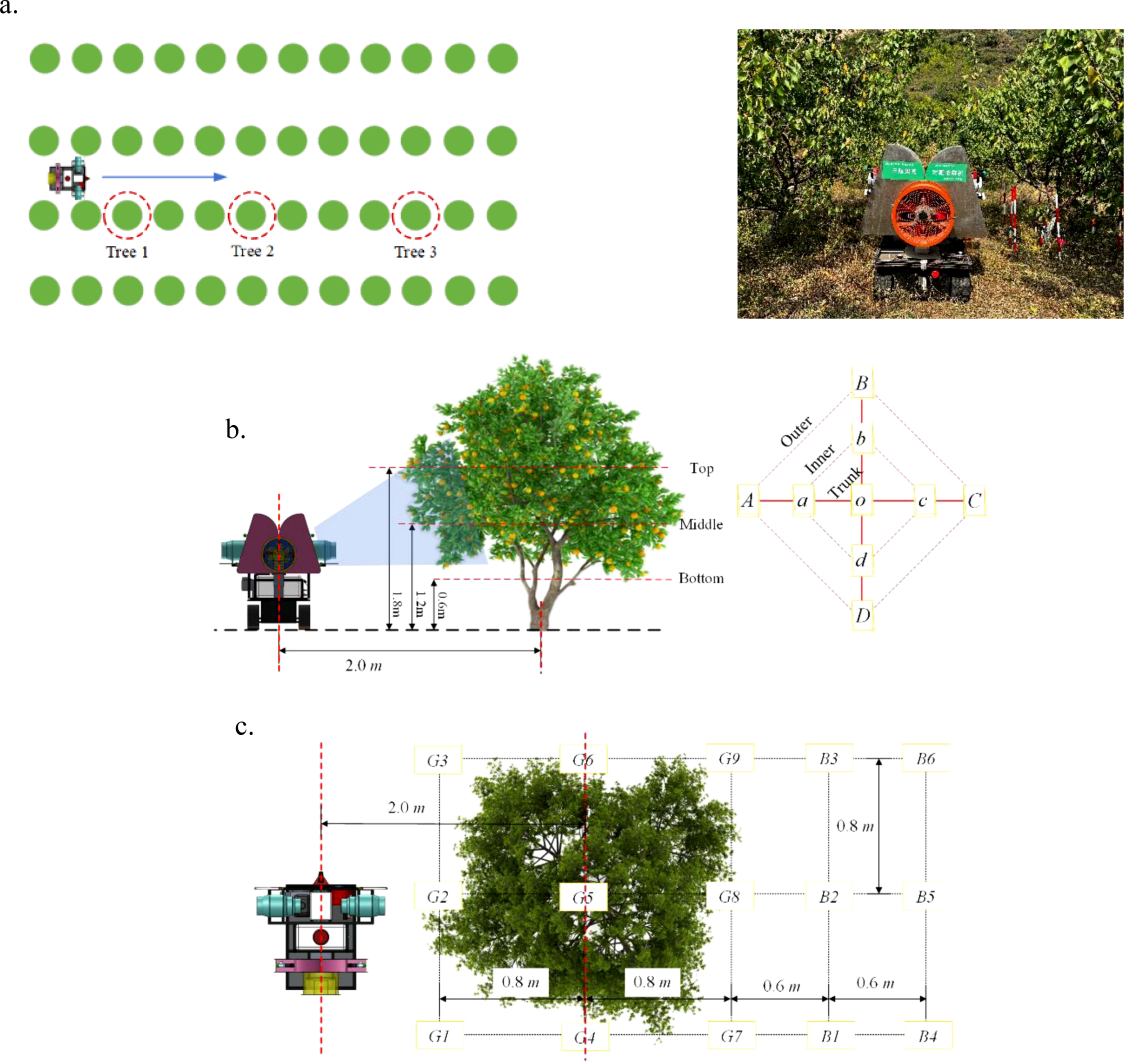
Schematic of the experiment scheme. **(a)** Selection of experimental trees and experimental scene. **(b)** Canopy sampling point scheme. **(c)** Ground sampling point scheme.

The experiment was conducted based on the standards issued by the Standardization Administration of China, including NY/T 992-2006 “The operation quality for air-assisted orchard sprayer” and JB/T 9782-2014 “Equipment for crop protection-General test methods”. The spray test should be carried out under environmental conditions with no rainfall, minimal dew, ambient temperatures between 5°C and 32°C and wind speeds not exceeding 3.5 m/s (below a light breeze). The wind speed and volume meter, AR856 produced by Shenzhen Franken Electronics Co., Ltd., and temperature and humidity meter produced by Deloitte Group Co., Ltd. were used to monitor and record meteorological parameters such as wind speed, wind direction, temperature and humidity. In the experiment, the temperature was 22°C, the humidity was 38%, and the average wind speed was 1.8 m/s. A 20 m × 50 m (width × length) area was selected as the experiment area, and three experimental trees were chosen in the area, away from the row ends. These trees were labeled as Tree 1, Tree 2, and Tree 3 (**Figure 6a**).

The canopy sampling point scheme is shown in **Figure 6b**. Each experimental fruit tree canopy was arranged with three layers of sampling points at heights of 1.8 m, 1.2 m, and 0.6 m, labeled as the top, middle, and bottom layers, respectively. Each layer had 9 sampling points: 4 on the outer side, 4 on the inner side, and 1 on the trunk. The distance between the outer and inner points (and between the inner points and the trunk) was approximately 0.6 m. The outer sampling points were labeled A, B, C, and D, the inner points are labeled a, b, c, and d, and the trunk point is labeled O. For ease of subsequent analysis, each sampling point was numbered, such as “A top,” “B middle,” and “D bottom,” etc. A and a were close to the sprayer, and the sprayer moved from D (d) towards B (b). During the experiment, water-sensitive paper (76 mm × 26 mm) was used to collect droplets. Two pieces of water-sensitive paper were placed at each sampling point on the canopy, clipped to the front and back of the leaves with a paper clip to collect droplets on both sides of the leaves. A total of 54 pieces of water-sensitive paper were arranged on each fruit tree canopy.

The ground sampling point scheme is shown in **Figure 6c**. Below the canopy of each experimental fruit tree, a 3×3 (row × column) grid of sampling points (9 points in total) was arranged, labeled as G1-G9. Points G1-G3 were located close to the sprayer. The distance between points was 0.8 m (both row-to-row and column-to-column), and the sampling layer was 0.3 m above the ground. At each sampling point, one piece of water-sensitive paper was placed with the front facing up to collect the droplets that have fallen to the ground. Behind each experimental fruit tree canopy, a 3×2 (row × column) grid of sampling points was arranged, labeled as B1-B6. Points B1-B3 were close to the canopy, and the distances between the tree trunk and the two columns of sampling points were 1.4 m and 2.0 m, respectively. The sampling layer was 0.3 m above the ground. At each sampling point, one piece of water-sensitive paper was placed with the front facing up to collect the droplets lost behind the canopy. The water-sensitive papers at the ground sampling points were fixed to the marker poles using binder clips, with a total of 15 pieces of water-sensitive paper arranged on the ground for each fruit tree.

The experiment was divided into single-sided spraying tests and double-sided spraying tests. The single-sided spraying test was conducted according to the previously described experimental scheme, while in the double-sided spraying test, only 9 ground sampling points (G1-G9) were arranged, with other conditions being the same as in the single-sided spraying test. To ensure consistent sprayer speed, the navigation walking mode was used during the experiment. The sprayer’s navigation speed was set to 0.5 m/s, and the spraying pressure was set to 0.5 MPa.

To verify the spraying effect of the target-variable sprayer, the sprayer was set to target-variable spraying mode (TV) and non-target and invariant variable mode (NTIV), with the spraying angle fixed at 15° in NTIV mode. For each experimental tree, the experiments were carried out in the TV mode sequence followed by the NTIV mode. The main experimental process is as follows:

- Mark the starting and ending points. Measure a distance of 20 m from the starting point using tape measure, which serves as the travel distance for the sprayer, positioning the experimental tree at the 10 m mark;
- Arrangement of water-sensitive paper at the canopy and ground sampling points according to the experimental scheme;
- Park the sprayer at the starting point, measure the liquid level in the tank, and record it;
- Start the sprayer and turn on the pump and fan. In TV mode, switch on the target-variable control, and in NTIV mode, remotely turn on the solenoid valve and proportional valve;
- After the sprayer runs for 5 seconds, turn on the automatic navigation switch to begin the spraying test;
- After reaching the endpoint, turn off the pump and fan, disable the automatic navigation switch and the target-variable switch in TV mode. In NTIV mode, remotely turn off the solenoid valve and proportional valve;
- Drive the sprayer back to the starting point, measure the liquid level in the tank;
- Wait for 15 minutes, collect the water-sensitive papers, and place them in sealed bags;
- Repeat steps II to VIII in the sequence of TV mode followed by NTIV mode;
- After completing the experiment on fruit tree 1, experiment on fruit trees 2 and 3 following steps I to IX.

### 2.5 Methods for evaluating spraying effects

To prevent contamination of the water-sensitive paper, the water-sensitive paper was collected and promptly scanned into JPG images using an EPSON scanner. The resolution of the image was 600 dpi. Then, the USDA Deposit Scan™ software was used to read the water-sensitive paper’s droplet coverage (Coverage, %), number of droplets (deposits, drops/cm²), and deposition (Deposition, μL/cm²). The data were saved in an Excel file, this study used software such as Excel 2021 and Origin 2019 to analyze the data.

The spraying effect of the target-variable sprayer is analyzed from aspects such as canopy droplet deposition (number of droplets), ground loss (deposition), and water consumption. To minimize the impact of environmental factors, the data from the water-sensitive papers at the sampling points of the three test trees were first averaged. Then, the coefficient of variation was used to measure the spray uniformity between the outer and inner layers of the canopy and between the top, middle, and bottom layers. The calculation method for the coefficient of variation is shown in Equation (10):


(10) $$C_{V}=\frac{\sigma }{\mu }\times 100\%$$

where *C_V_* is the coefficient of variation of the sample, *μ* is the mean of the sample and *σ* is the standard deviation of the sample.

For convenience in subsequent analysis, the distribution relationship between the top, middle, and bottom layers was referred to as the longitudinal distribution, and the relationship between the outer layer, inner layer, and trunk was referred to as the lateral distribution. The calculation method of the coefficient of variation of longitudinal distribution: first average the number of droplets at all sampling points in each layer, then calculate the average and standard deviation of the number of droplets in the three layers after averaging, and finally calculate according to Equation (10) Longitudinal distribution coefficient of variation. The calculation method of the lateral distribution variation coefficient: Calculate the mean and standard deviation of the 9 sampling points in each layer, and then calculate the lateral distribution variation coefficient according to Equation (10).

The method for calculating the water consumption is shown in Equation (11):


(11) $$V_{w}=L_{b}\times W_{b}\times \left(H_{bs}-H_{be}\right)$$

where *V_w_* is the water consumption, *L_b_* is the length of the tank, *W_b_* is the width of the tank, *H_bs_* is the liquid level height at the start of spraying, and *H_be_* is the liquid level height at the completion of spraying.

The calculation of ground loss was based on the deposition (Deposition, μL/cm²) the ground water-sensitive papers (G1-G9, B1-B6). For the convenience of analysis, a plane rectangular coordinate system was constructed with the tree trunk (G5 sampling point) as the coordinate origin. The direction of the sprayer was the positive direction of the X-axis, and the right direction perpendicular to the X-axis was the positive direction of the Y-axis (spraying direction). The sampling points on the ground can be located by coordinates, for example, the coordinates of point G1 were (-0.8, -0.8), the coordinates of point G5 were (0, 0), and the coordinates of point B6 were (2.0, 0.8).

## 3 Results and discussion

### 3.1 Spray flow rate calibration results

**Figure 7:**
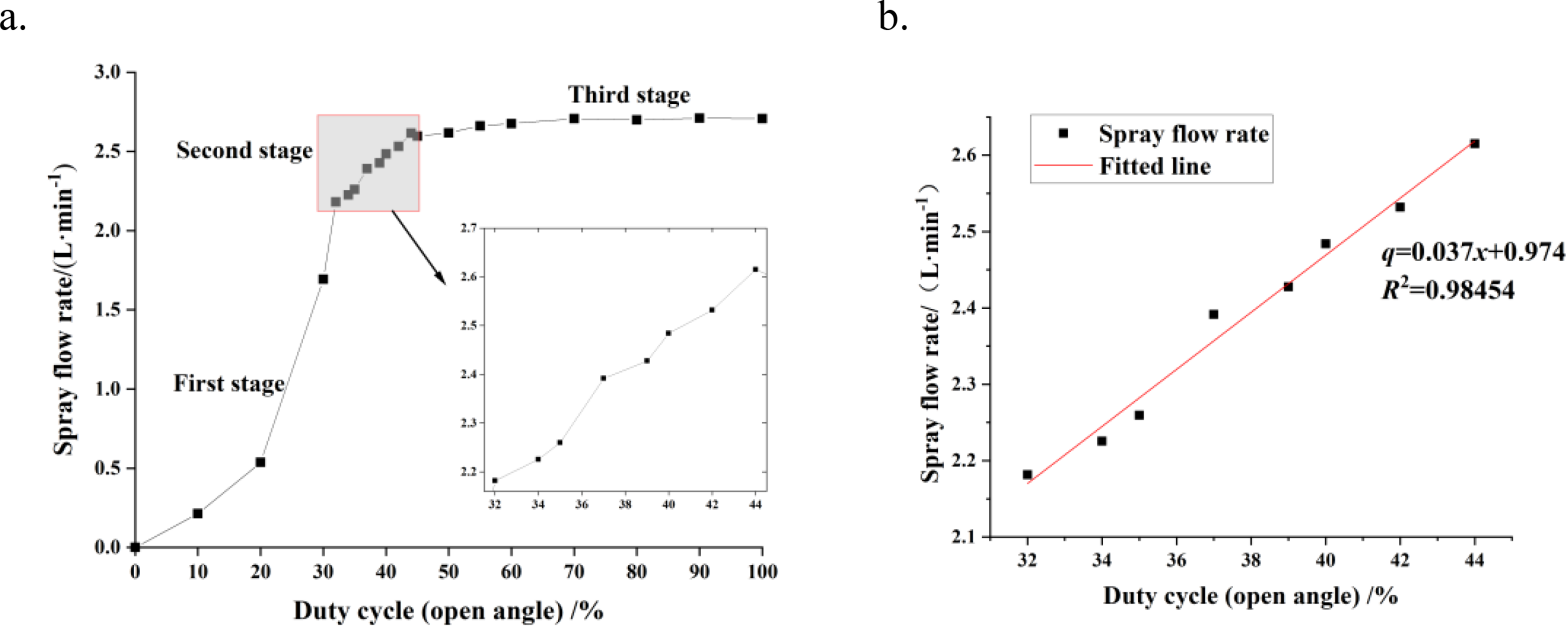
Calibration and fitting of spray flow rate. **(a)** Calibration results of spray flow rate and duty cycle. **(b)** Fitting of spray flow rate and duty cycle.


The calibration results of duty cycle and spray flow rate are shown in **Figure 7a**. As the duty cycle signal increases, the spray flow rate first increases rapidly (first stage), then grows steadily (second stage), and finally tends to be stable (third stage). Since the spray flow rate increases too rapidly in the first stage, making control relatively difficult, and the adjustment of the duty cycle in the third stage has little impact on the spray flow rate, the second stage, where the duty cycle ranges from 32% to 44%, was selected for further analysis to facilitate the construction of the subsequent spray dosage control model. The fitting results for this stage are shown in **Figure 7b**. The functional relationship between the spray flow rate *q* and the duty cycle *x* follows Equation (12), with a coefficient of determination *R* ^2^ = 0.98454.

(12) q=0.037x+0.974

Combining Equation (9) and Equation (12), the relationship between FAVD and the duty cycle can be expressed by Equation (13):


(13) $$x=\frac{2.27\times 10^{6}\times kv\;\rho_{\;favd}\pi C_{v}^{3}}{\left(1-\delta L_{d}\right)PL_{v}}-26.32$$

Therefore, considering the actual spraying requirements and the characteristics of the small target-variable sprayer, the variable spraying control model based on PWM duty cycle *x* is shown in Equation (14):


(14) $$x=\left\{ \begin{array}{l} \begin{array}{cc} 32, & 0<x<32 \end{array}\\ \begin{array}{cc} \frac{2.27\times 10^{6}\times kv\;\rho_{\;favd}\pi C_{v}^{3}}{\left(1-\delta L_{d}\right)PL_{v}}-26.32, & 32\leq x\leq 44 \end{array}\\ \begin{array}{cc} 44, & 44<x<100 \end{array} \end{array}\right.$$

### 3.2 Canopy droplet distribution

#### 3.2.1 Results of single-sided spraying canopy droplet distribution

(1) Analysis of the longitudinal distribution of orchard canopy

**Figure 8:**
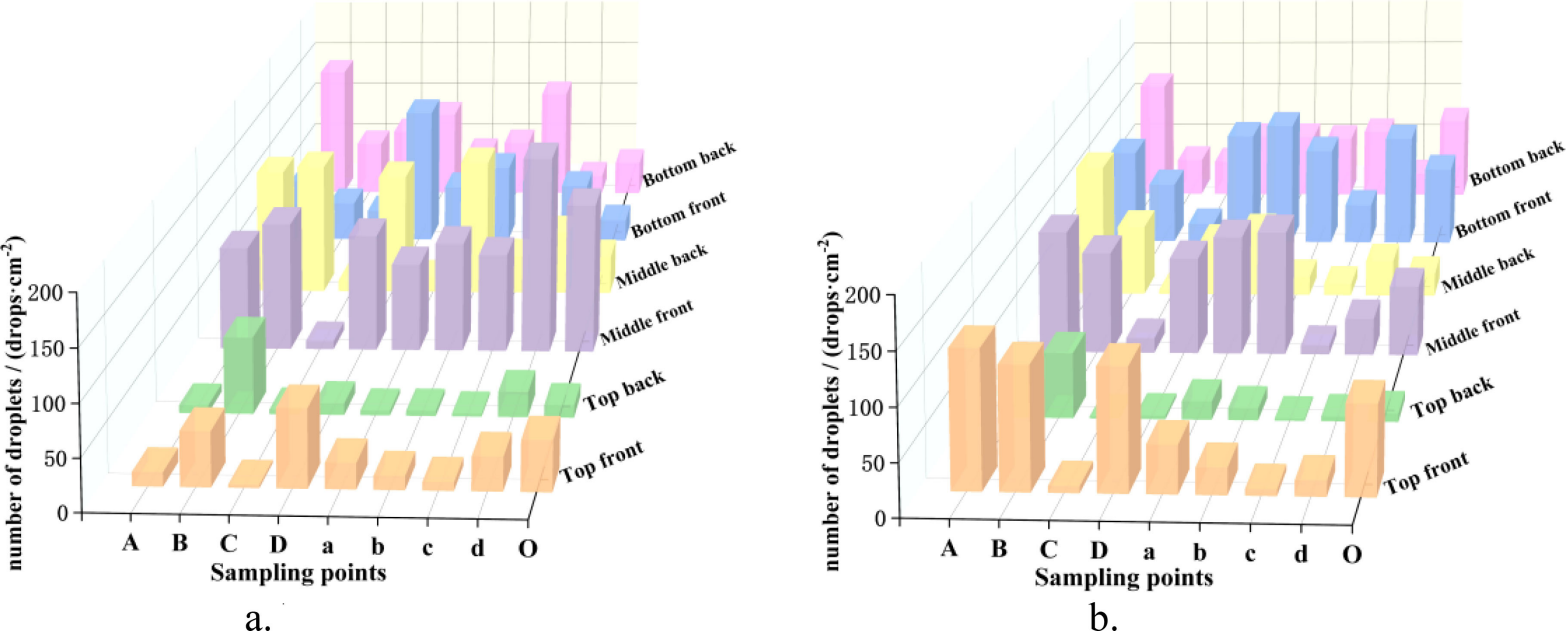
Distribution of number of droplets at sampling points. **(a)** NTIV Mode. **(b)** TV Mode.

The distribution of the number of droplets at each sampling points in the canopy under different spraying modes is shown in **Figure 8** and **Table 2**. Based on the droplet distribution and longitudinal coefficient of variation, the TV mode was more uniform in the longitudinal distribution of the canopy than the NTIV mode.

**Table 2:** Canopy average number of droplets and longitudinal coefficient of variation.

Mode	Position	Average number of droplets/(drops·cm^-2^)		Longitudinal coefficient of variation	
		Front	Back	Front	Back
NTIV	Top Layer	30.10	16.60	42.85%	49.95%
	Middle Layer	111.54	78.04		
	Bottom Layer	64.29	74.51		
TV	Top Layer	61.10	17.19	13.90%	46.15%
	Middle Layer	77.08	47.85		
	Bottom Layer	86.28	66.07		

In the NTIV mode, the longitudinal coefficients of variation for the front and back of the leaf were 42.85% and 49.95%, respectively. The average number of droplets on the front of the leaf in the top, middle, and bottom layers were 30.10 drops/cm², 111.54 drops/cm², and 64.29 drops/cm², respectively. Although all of these meet the minimum spraying requirement (25/cm²), there was considerable variation in the top layer, with only points B, D, d, and O meeting the requirement, while other points do not reach the spraying standard. The average number of droplets on the back of the leaf were 16.60 drops/cm², 78.04 drops/cm², and 74.51 drops/cm² for the top, middle, and bottom layers, respectively. In the middle and bottom layers, all points except C middle and d bottom meet the spraying requirement, whereas in the top layer, only point B meets the spraying requirement.

In the TV mode, the longitudinal coefficients of variation for the front and back of the leaf were 13.90% and 46.15%, respectively. The average number of droplets on the front of the leaf in the top, middle, and bottom layers were 61.10 drops/cm², 77.08 drops/cm², and 86.28 drops/cm², respectively. Except for points C and c, which were far from the sprayer, all other points on the front of the leaf meet the spraying requirements. Compared to the NTIV mode, the TV mode not only meets the spraying requirements but also reduces the average number of droplets while significantly improving uniformity. For the middle and bottom layers, except for the points far from the sprayer, the number of droplets on the back of the leaf has improved in terms of spraying effectiveness at all other points.

(2) Analysis of the lateral distribution of canopy

**Figure 9:**
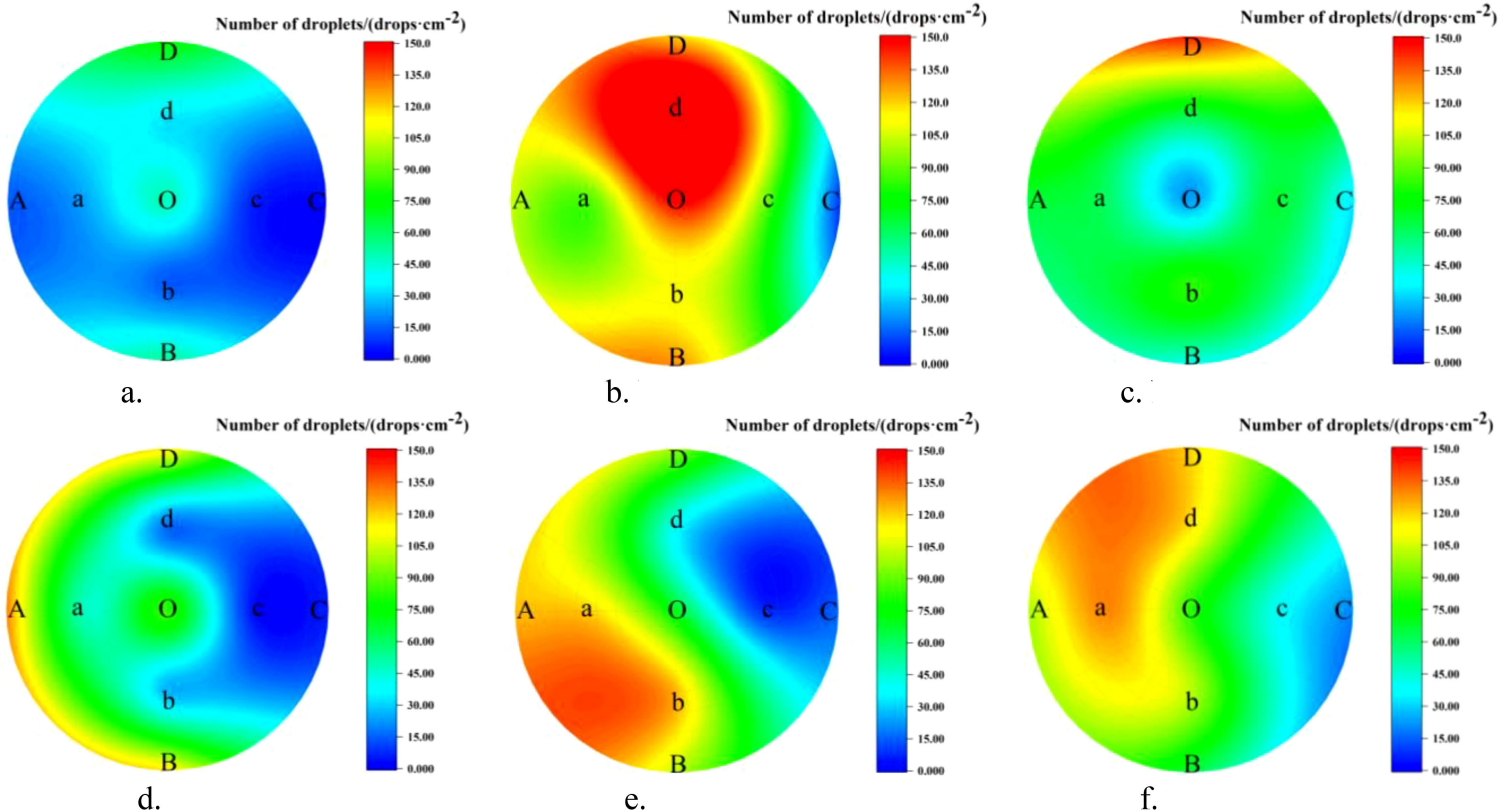
Lateral distribution of canopy under different modes. **(a)** NTIV top layer. **(b)** NTIV middle layer. **(c)** NTIV bottom layer. **(d)** TV top layer. **(e)** TV middle layer. **(f)** TV bottom layer.

The droplet distribution on the leaves in the top, middle, and bottom layers of the canopy, as well as the lateral coefficients of variation under the two spraying modes, are shown in **Figure 9** and **Table 3**.

**Table 3:** Lateral coefficient of variation of the leaves.

Mode	Top Layer	Middle Layer	Bottom Layer
NTIV	75.20%	42.80%	52.08%
TV	79.70%	56.50%	41.09%

As shown in **Figure 9** and **Table 3**, a-c represents the number of droplets distribution under the NTIV mode, with lateral coefficients of variation of 75.20%, 42.80%, and 52.08%, respectively. Overall, the number of droplets in the middle and bottom layers were significantly higher than in the top layer, with most of the droplets in the top layer not reaching the minimum spraying requirement (25 drops/cm²). d-f represents the number of droplets distribution under the TV mode, with lateral coefficients of variation of 79.70%, 56.50%, and 41.09%, respectively. The droplets in the top layer show some improvement compared to the NTIV mode, but the performance at points C and c, which are farther from the sprayer, did not show significant improvement. The stratification phenomenon in the middle and bottom layers was more obvious. The farther away from the sprayer, the fewer the number of droplets. Except for C and c, which fail to meet the requirements, the others can meet the spraying needs.

From the perspective of the variation coefficient, the uniformity of droplet distribution in each layer has improved under the TV mode, with the improvement in the bottom layer being particularly noticeable. The NTIV mode exhibits clear signs of over-spraying, such as 197.07 drops/cm² at point d middle and 149.93 drops/cm² at point O middle. The TV mode shows improvement, with droplet counts below 100 drops/cm² at all points except for a few closest to the sprayer.

#### 3.2.2 Results of canopy droplet distribution in double-sided spraying

##### 3.2.2.1 Analysis of the longitudinal distribution of canopy droplets

**Figure 10:**
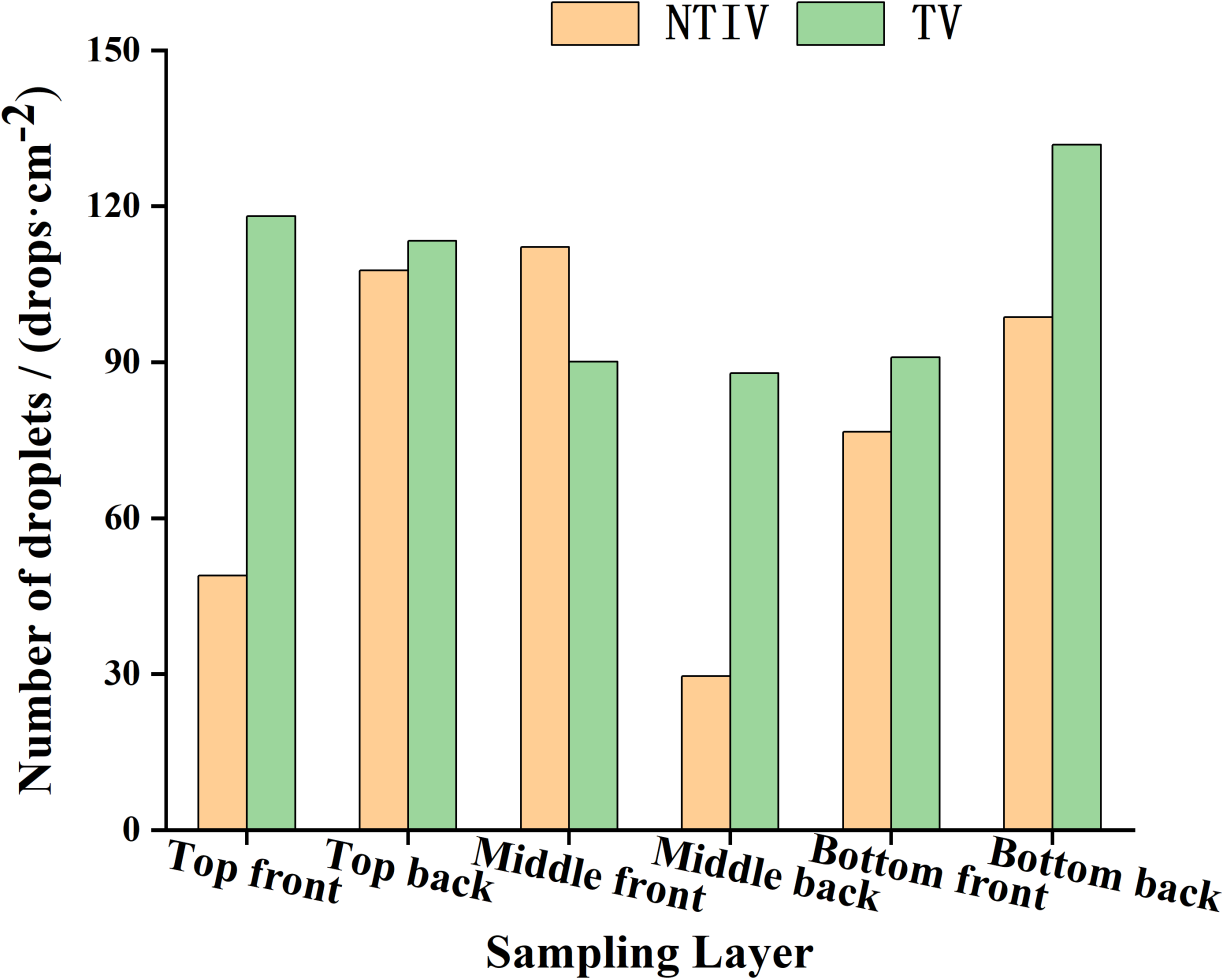
Droplet distribution in each layer under double-sided spraying.

The distribution of the number of droplets in the canopy at each layer and the longitudinal distribution coefficient of variation are shown in **Figure 10** and **Table 4**.

**Table 4:** Longitudinal coefficient of variation of the canopy in double-sided spraying.

Mode	Position	Average number of droplets/(drops·cm^-2^)		Longitudinal coefficient of variation	
		Front	Back	Front	Back
NTIV	Top Layer	48.91	29.62	32.15%	42.14%
	Middle Layer	107.63	40.76		
	Bottom Layer	112.1	87.90		
TV	Top Layer	118.04	87.90	11.42%	19.35%
	Middle Layer	113.3	90.93		
	Bottom Layer	90.04	131.84		

In both the NTIV and TV modes, the average number of droplets on the front and back of the leaves in each layer exceed 25 drops/cm². The sampling point with the least number of droplets was the top layer of the NTIV mode (29 drops/cm^2^), which also met the requirements for spraying. In the NTIV mode, the number of droplets on the front and back of the leaves increase gradually from the top to the bottom layers, and the coefficients of variation on the front and back sides were 32.15% and 42.14%, respectively, which were smaller than the coefficient of variation of single-sided spraying, and the uniformity was improved. In the TV mode, the variation coefficients on the front and back were 11.42% and 19.35%, respectively, both of which were smaller than those in the NTIV mode, indicated better spraying uniformity. Compared to single-sided spraying, double-sided spraying increased the number of droplets in the top canopy and on the back of the leaves and also improved uniformity. Except for a few points with lower droplets, most sampling points met the spraying requirements.

##### 3.2.2.2 Analysis of the lateral distribution of canopy droplets

**Figure 11:**
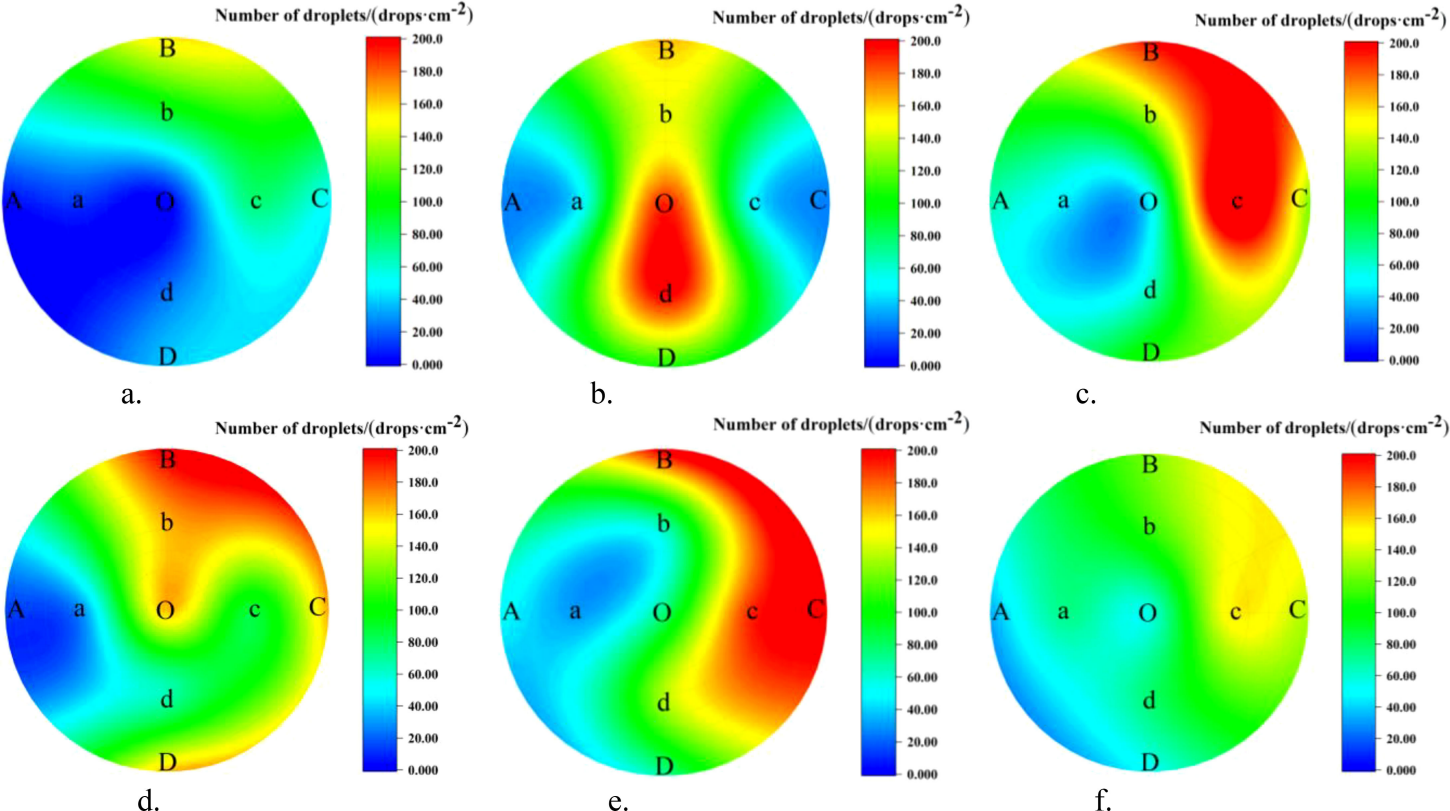
Lateral distribution of droplet in the canopy under double-sided spraying. **(a)** NTIV top layer. **(b)** NTIV middle layer. **(c)** NTIV bottom layer. **(d)** TV top layer. **(e)** TV middle layer. **(f)** TV bottom layer.

The droplet distribution in the top, middle, and bottom layers of the canopy, along with the lateral coefficients of variation, are shown in **Figure 11** and **Table 5**. In the NTIV mode, the lateral coefficients of variation for the top, middle, and bottom layers are 96.19%, 62.69%, and 57.19%, respectively, with the variation coefficient gradually decreasing from top to bottom. The number of droplets on the leaves was less than 25 drops/cm^2^ only at points A and O in the top layer, while the other points were all higher than 25 drops/cm^2^. However, the differences between the sampling points were relatively large, and the sampling point with the largest number of droplets was 44.37 times that of the smallest. In the TV mode, the lateral coefficients of variation for the top, middle, and bottom layers were 55.27%, 58.80%, and 43.15%, respectively. The uniformity in each layer was better than in the NTIV mode, with only point A in the top layer failing to meet the requirement. Compared to single-sided spraying, double-sided spraying increased the number of droplets at sampling points in all layers of the canopy.

**Table 5:** Lateral coefficients of variation of the canopy under double-sided spraying.

Mode	Top Layer	Middle Layer	Bottom Layer
NTIV	96.19%	62.69%	57.19%
TV	55.27%	58.80%	43.15%

### 3.3 Ground loss

#### 3.3.1 Analysis of ground loss in single-sided spraying

**Figure 12:**
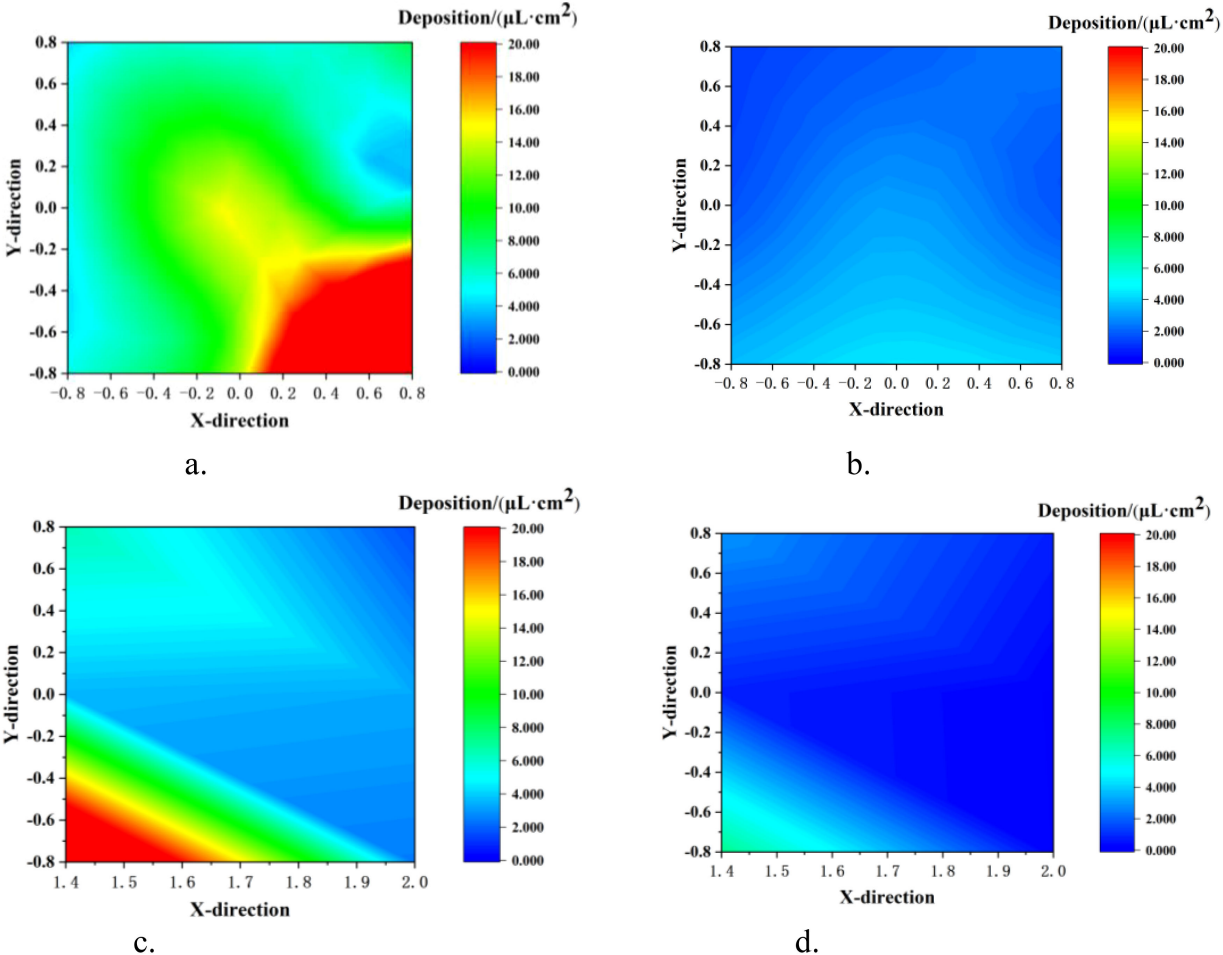
Ground loss in single-sided spraying. **(a)** NTIV Ground. **(b)** TV Ground. **(c)** NTIV behind-Canopy. **(d)** TV behind-Canopy.

The ground loss under different modes is shown in **Figure 12**. It can be seen intuitively from the figure that the droplet deposition in the TV mode was significantly reduced both on the canopy ground and behind-canopy. The average ground deposition in the two modes was 13.31 μL/cm² (NTIV mode) and 2.69 μL/cm² (TV mode), while the average behind-canopy deposition was 7.61 μL/cm² (NTIV mode) and 2.01 μL/cm² (TV mode). In the NTIV mode, point G7 (56.55 μL/cm²) and point B1 (28.31 μL/cm²) have the highest droplet deposition. Compared to the NTIV mode, the TV mode can reduce ground loss by 79.78%, and behind-canopy loss by 73.54%. According to previous test results (Jiang et al., 2022, Jiang et al., 2023), the average ground loss of tower air-assisted sprayers was 117.69 μL/cm², and compared to this, the TV mode can reduce ground loss by 97.71%. Therefore, adjusting the spray angle and dosage based on FAVD can effectively reduce droplets loss on the ground and behind-canopy.

#### 3.3.2 Analysis of ground loss in double-sided spraying

**Figure 13:**
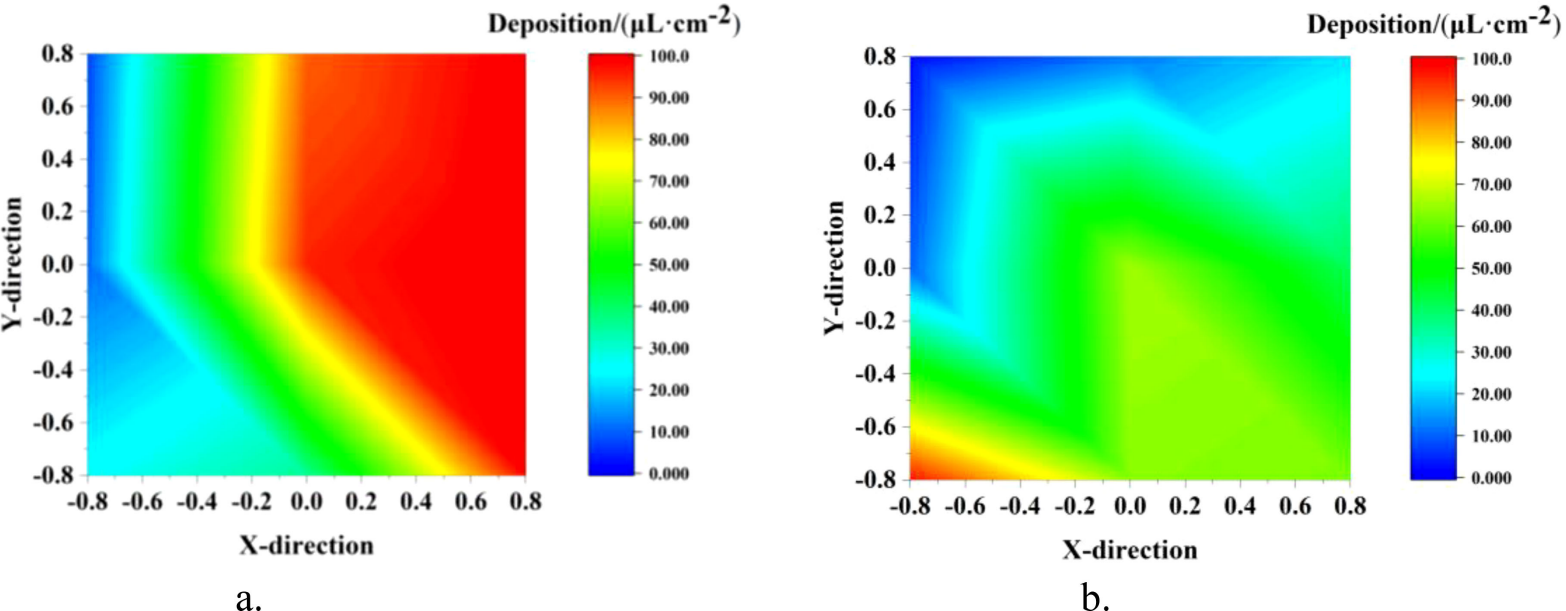
Ground loss in double-sided spraying. **(a)** NTIV Ground. **(b)** TV Ground.

The ground loss under different modes is shown in **Figure 13**, The average ground deposition in the two modes were 62.82 μL/cm² (NTIV mode) and 41.80 μL/cm² (TV mode). Compared to the NTIV mode, the TV mode can reduce ground loss by about 33.46%. Compared to tower air-assisted sprayers, the TV mode in double-sided spraying can reduce ground loss by about 64.49%. Compared to single-sided spraying, double-sided spraying results in increased ground loss. However, adjusting the spray angle and dosage based on canopy characteristics can effectively reduce the droplet loss on the ground.

### 3.4 Water Consumption

**Figure 14:**
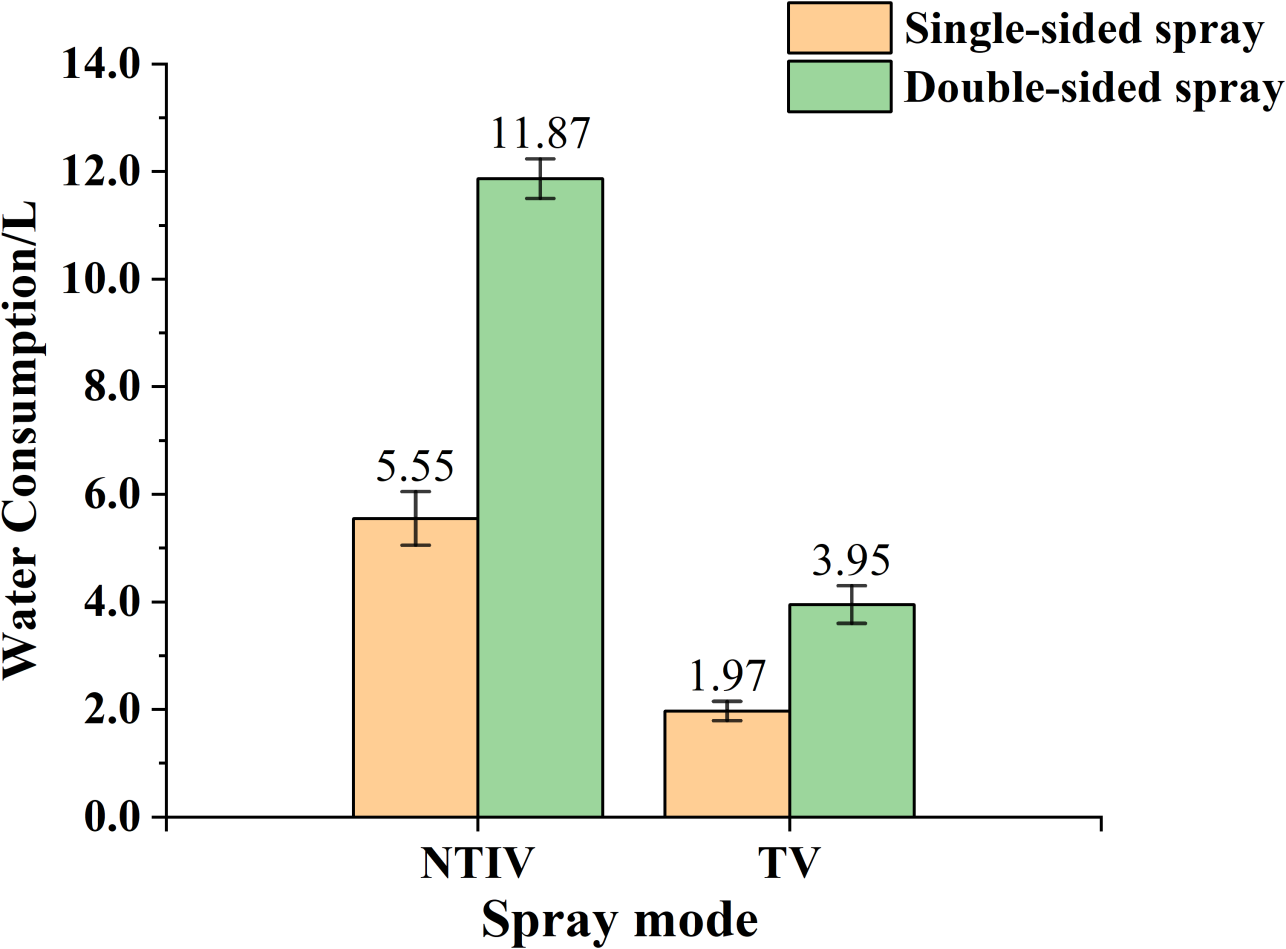
Water consumption in different modes.

As shown in **Figure 14**, the water consumption was calculated using Equation (11), and the average water consumption of three experimental trees under the same operational mode was taken. During single-sided spraying, the water consumption for the NTIV and TV modes was 5.55 L and 1.97 L, respectively. Under the same operating distance conditions, the TV mode saved 64.50% of water compared to the NTIV mode. For double-sided spraying, the water consumption for NTIV and TV modes was 11.87 L and 3.95 L, respectively, with the TV mode saving 66.72%. This demonstrates that target-variable spraying based on the FAVD of the canopy can effectively reduce water consumption.

### 3.5 Discussions

This study takes the low-dose pesticide spray standard for fruit trees as the starting point. A spray flow rate control model based on FAVD was constructed. The model adjusts the PWM duty cycle according to the change of FAVD to achieve variable spraying. In response to the practical needs for inter-row accessibility and quick transitions in orchards (especially those with large canopies and dense inter-row coverage), a small air-assisted target-variable sprayer based on FAVD was developed. This system enables flexible switching between control and spraying modes according to the actual conditions of the orchard, providing a technical reference for the optimization of air-assisted spray equipment in orchards.

However, there are still some shortcomings:

- The spray flow rate control model based on FAVD proposed in this study can realize variable spraying. Experimental results show that the uniformity of canopy droplet distribution, reduction of ground loss, and water saving. However, it did not fundamentally solve the issue of low spray deposition on the back of leaves. The variation coefficient between the front and back of leaves remains large. The likely reason was that the density of trichomes and surface roughness differ between the two sides, which affects droplet adhesion. Future research could focus on understanding how these characteristics influence droplet adherence to further improve pesticide efficiency and reduce loss.
- The small target-variable sprayer developed in this study can spray both sides of the canopy simultaneously. In the experiments, only the excitation fan on one side of the sprayer was turned on, that is, FAVD estimation and spray were only carried out on one side of the canopy. When the excitation fans on both sides were turned on at the same time, the problem of excitation audio segmentation of the canopies on both sides was not involved in this study, which can be used as the focus and difficulty of the next study.

## 4 Conclusion

This study focused on large-canopy fruit trees and developed a small target-variable sprayer based on FAVD. A spray flow rate control model was designed based on FAVD, and orchard experiments were conducted, and the main conclusions are as follows:

1. A small target-variable sprayer was developed, and a spray flow rate control model based on FAVD was constructed. In response to the needs of inter-row passability and convenient transfer in orchards, based on actual orchard surveys, the overall scheme and key components of the sprayer were analyzed, a FAVD detection system was designed, a target system and an automatic navigation system were integrated, the control system was developed, and the prototype was trial-produced. The relationship between the PWM duty cycle signal and the spray flow rate was determined through calibration. A spray flow control model based on FAVD was constructed, which can achieve variable spraying by adjusting the duty cycle signal for canopies with different FAVDs.
2. Orchard experiments were conducted, and the results showed that in TV mode, when spraying on both sides, the longitudinal coefficient of variation was 11.42%, and the lateral coefficients of variation were 55.27% (top layer), 58.80% (middle layer), and 43.15% (bottom layer). Compared with the NTIV mode, when spraying on one side, the ground loss was reduced by 79.78%, the behind-canopy loss was reduced by about 73.54%, the water consumption was saved by 64.50%. The ground loss of double-sided spraying was reduced by about 33.46%. Overall, TV mode showed advantages in terms of canopy droplet deposition uniformity, reduced ground runoff, and lower pesticide usage.

The results of this study can enhance droplet coverage in fruit tree canopies, meeting pest control requirements while reducing ground loss and environmental pollution. The developed small target-variable sprayer and the constructed spray flow control model based on FAVD can guide the optimization of precision orchard protection technologies and equipment. These findings provide valuable references for the development of precision spraying technologies in the context of “reduced pesticide use and application.”

## Data Availability

The raw data supporting the conclusions of this article will be made available by the authors, without undue reservation.

## References

[B1] Agricultural Machinery of Standardization Administration of China. (2006). NYT 992-2006: The operation quality for air-assisted orchard sprayer (Beijing: Ministry of Agriculture and Rural Affairs of the People’s Republic of China).

[B2] Cai J. Wang X. Gao Y. Yang S. Zhao C. (2019). Design and performance evaluation of a variable-rate orchard sprayer based on a laser-scanning sensor. Int. J. Agric. Biol. Eng. 12, 51–57. doi: 10.25165/j.ijabe.20191206.4174

[B3] Chen C. Jia Y. Zhang J. Yang L. Wang Y. Kang F.. (2024). Development of a 3D point cloud reconstruction-based apple canopy liquid sedimentation model. J. Cleaner Production 451, 142038. doi: 10.1016/j.jclepro.2024.142038

[B4] Dou H. Zhai C. Wang X. Zou W. Li Q. Chen L.. (2022). Design and experiment of the orchard target variable spraying control system based on LiDAR. Trans. CSAE 38, 11–21. doi: 10.11975/j.issn.1002-6819.2022.03.002

[B5] Gao G. Xiao K. Li J. (2018). Precision spraying model based on kinect sensor for orchard applications. Appl. Eng. Agric. 34, 291–298. doi: 10.13031/aea.12538

[B6] Gu C. Wang X. Wang X. Yang F. Zhai C. (2020). Research progress on variable-rate spraying technology in orchards. Appl. Eng. Agric. 36, 927–942. doi: 10.13031/aea.14201

[B7] Gu C. Zhai C. Wang X. Wang S. (2021). CMPC: an innovative Lidar-based method to estimate tree canopy meshing-profile volumes for orchard target-oriented spray. Sensors 21, 4252 doi: 10.3390/s21124252 34205819 PMC8235039

[B8] Hoevar M. Širok B. Jejic V. Godeša T. Lešnik M. Stajnko D. (2010). Design and testing of an automated system for targeted spraying in orchards. J. Plant Dis. Prot. 117, 71–79. doi: 10.1007/BF03356338

[B9] Jiang H. Bai P. Liu L. Deng X. Song J. Zhang X.. (2016). Caterpillar self-propelled and air-assisted orchard sprayer with automatic target spray system. Trans. Chin. Soc. Agric. Machinery 47, 189–195. doi: 10.6041/j.issn.1000-1298.2016.S0.029

[B10] Jiang S. Li W. Yang S. Zheng Y. Tan Y. Xu J.. (2023). Factors affecting droplet loss behind canopies with air-assisted sprayers used for fruit trees. Agronomy 13, 375. doi: 10.3390/agronomy13020375

[B11] Jiang S. Yang S. Xu J. Zheng W. Liu Y. X.. (2022). Wind field and droplet coverage characteristics of air-assisted sprayer in mango-tree canopies. Pest Manage. Sci. 78, 4892–4904. doi: 10.1002/ps.v78.11 36053879

[B12] Li W. Jiang S. Yang S. Feng H. Liu W. Zheng Y.. (2024). Leaf-density estimation for fruit-tree canopy based on wind-excited audio. J. Field robotics 41, 1469–1479. doi: 10.1002/rob.22336

[B13] Li S. Li J. Yu S. Wang P. Liu H. Yang X. (2023). Anti-drift technology Progress of plant protection applied to orchards: a review[J. Agronomy 13, 2679 doi: 10.3390/agronomy13112679

[B14] Liao J. Zang Y. Luo X. Zhou Z. Zang Y. Wang P.. (2020). The relations of leaf area index with the spray quality and efficacy of cotton defoliant spraying using unmanned aerial systems (UASs). Comput. Electron. Agric. 169, 105228. doi: 10.1016/j.compag.2020.105228

[B15] Liu H. Du Z. Shen Y. Du W. Zhang X. (2024). Development and evaluation of an intelligent multivariable spraying robot for orchards and nurseries. Comput. Electron. Agric. 222, 109056. doi: 10.1016/j.compag.2024.109056

[B16] Liu L. Liu Y. He X. Liu W. (2022). Precision variable-rate spraying robot by using single 3d lidar in orchards. Agronomy 12, 2509 doi: 10.3390/agronomy12102509

[B17] Mahmud M. Zahid A. He L. Choi D. Krawczyk G. Zhu H.. (2021). Development of a LiDAR-guided section-based tree canopy density measurement system for precision spray applications. Comput. Electron. Agric. 182, 106053. doi: 10.1016/j.compag.2021.106053

[B18] Manandhar A. Zhu H. Ozkan E. Shah A. (2020). Techno-economic impacts of using a laser-guided variable-rate spraying system to retrofit conventional constant-rate sprayers. Precis. Agric. 21, 1156–1171. doi: 10.1007/s11119-020-09712-8

[B19] Meshram A. Vanalkar A. Kalambe K. Badar A. (2022). Pesticide spraying robot for precision agriculture: A categorical literature review and future trends. J. Field Robotics 39, 153–171. doi: 10.1002/rob.22043

[B20] Narváez F. Pedregal J. Prieto P. Torres-Torriti M. Cheein F. (2016). LiDAR and thermal images fusion for ground-based 3D characterisation of fruit trees. Biosyst. Eng. 151, 479–494. doi: 10.1016/j.biosystemseng.2016.10.012

[B21] Salcedo R. Zhu H. Zhang Z. Wei Z. Chen L. Ozkan E.. (2020). Foliar deposition and coverage on young apple trees with PWM-controlled spray systems. Comput. Electron. Agric. 178, 105794. doi: 10.1016/j.compag.2020.105794

[B22] Sun C. Liu C. (2019). Construction and application of droplet canopy penetration model for air-assisted spraying pattern. Trans. CSAE 35, 25–32. doi: 10.11975/j.issn.1002-6819.2019.15.004

[B23] Sun D. Liu W. Luo R. Zhan X. Chen Z. Wei T.. (2022). Monocular vision for variable spray control system. Int. J. Agric. Biol. Eng. 15, 206–215. doi: 10.25165/j.ijabe.20221506.7646

[B24] Wang Y. (2018). Study on precision spray technology for barrier and atomization model for nozzles (Beijing Forestry University). doi: 10.26949/d.cnki.gblyu.2018.000002

[B25] Wei Z. Xue X. Salcedo R. Zhane Z. Gil E. Sun Y.. (2023). Key technologies for an orchard variable-rate sprayer: Current status and future prospects. Agronomy 13, 59. doi: 10.3390/agronomy13010059

[B26] Xiao K. Ma Y. Gao G. (2017). An intelligent precision orchard pesticide spray technique based on the depth-of-field extraction algorithm. Comput. Electron. Agric. 133, 30–36. doi: 10.1016/j.compag.2016.12.002

[B27] Xue X. Xu X. Li Z. Hong T. Xie J. Chen J.. (2020). Design and test of variable spray model based on leaf wall area in orchards. Trans. CSAE 36, 16–22. doi: 10.11975/j.issn.1002-6819.2020.02.003

[B28] Zeng L. Feng J. He L. (2020). Semantic segmentation of sparse 3D point cloud based on geometrical features for trellis-structured apple orchard. Biosyst. Eng. 196, 46–55. doi: 10.1016/j.biosystemseng.2020.05.015

